# Ins and Outs of Multipartite Positive-Strand RNA Plant Viruses: Packaging versus Systemic Spread

**DOI:** 10.3390/v8080228

**Published:** 2016-08-18

**Authors:** Mattia Dall’Ara, Claudio Ratti, Salah E. Bouzoubaa, David Gilmer

**Affiliations:** 1 Institut de Biologie Moléculaire des Plantes, Integrative Virology, CNRS UPR2367, Université de Strasbourg, 12 rue du Général Zimmer, 67084 Strasbourg, France; mattia.dallara5@unibo.it (M.D’A.); salah.bouzoubaa@ibmp-cnrs.unistra.fr (S.E.B.); 2 Dipartimento di Scienze Agrarie, Area Patologia Vegetale, Università di Bologna, Viale Fanin 40, 40127 Bologna, Italy

**Keywords:** phytovirus, segmented genome, genome integrity, systemic movement, RNA-RNA interaction

## Abstract

Viruses possessing a non-segmented genome require a specific recognition of their nucleic acid to ensure its protection in a capsid. A similar feature exists for viruses having a segmented genome, usually consisting of viral genomic segments joined together into one viral entity. While this appears as a rule for animal viruses, the majority of segmented plant viruses package their genomic segments individually. To ensure a productive infection, all viral particles and thereby all segments have to be present in the same cell. Progression of the virus within the plant requires as well a concerted genome preservation to avoid loss of function. In this review, we will discuss the “life aspects” of chosen phytoviruses and argue for the existence of RNA-RNA interactions that drive the preservation of viral genome integrity while the virus progresses in the plant.

## 1. Introduction

Preserving genome integrity is a key challenge for any organism, and viruses with an RNA-based genome are not excluded from this basic rule. Obligate parasites that have a monopartite genome mainly face recombination events that draw viral evolution. While viruses with segmented genomes also encounter similar evolutionary traits, another constraint applies to the maintenance of genome integrity. Indeed, all genomic segments should be available within the same cell and be transmitted from one cell or one organism to another. To do so, all genomic components must be packaged within the same viral particle. This is well exemplified by *Orthomyxoviridae* members where all genomic negative-stranded ribonucleoprotein (RNP) complexes are selectively assembled together within one enveloped viral particle [1], or *Bunyaviridae* [2] where all three segments are maintained together within the envelope. Similar examples can be drawn for double-stranded RNA viruses such as *Reoviridae* or the well-known *Cystoviridae* pseudomonas phage Ø6 that possesses three genomic double-stranded (ds)RNA segments within the same viral structure [3]. 

Viruses that possess a positive-stranded RNA genome are found in genera that infect bacteria (e.g., *Leviviridae* enterobacteria phage Qß), animals and insects (e.g., *Picornavirales* enterovirus C—namely poliovirus—and cricket paralysis virus) and also plants (e.g., *Virgaviridae*, tobacco mosaic virus). To date, distinguishing features of (+)-strand RNA viruses that infect bacteria, animals or insects reside in the nature of the viral genome that is limited to either one single-stranded (ss)RNA molecule or two genomic RNAs packaged within a unique icosahedral capsid (e.g., *Nodaviridae*, flock house virus). However, (+)-strand RNA phytoviruses with segmented genomes range between two to five positive-strand RNA molecules and such so-called multipartite viruses possess either an icosahedral (sometimes bacilliform) or helical shape. A large majority of these phytoviruses have distinctive features compared to the aforementioned animal (+)-strand RNA viruses, as each segment can be individually packaged as ribonucleoprotein complexes within a helical structure. Icosahedral phytoviruses with a segmented positive-sense RNA genome either package single segment species in each capsid or a combination of segments within the limits of physical size constraints. A.L.N. Rao has reviewed mechanisms driving genome packaging of spherical plant RNA viruses [4] while Solovyev and Makarov recently focused on plant viruses with helical capsids [5].

**Figure 1:**
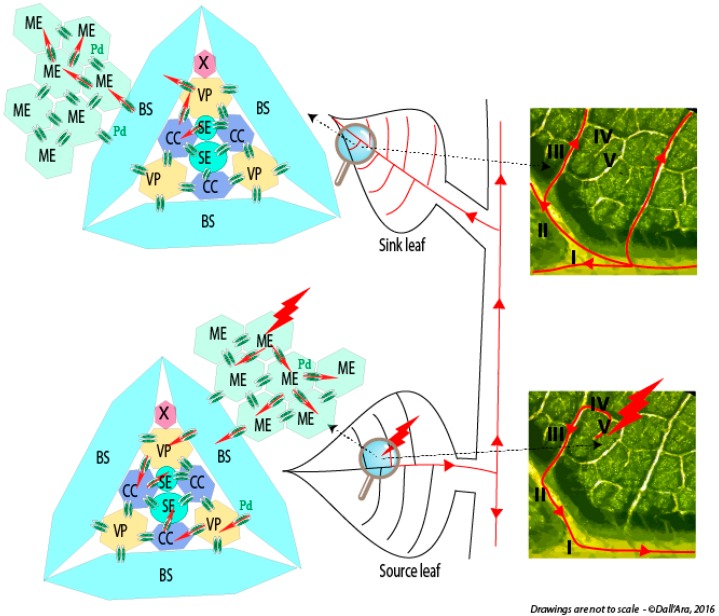
Schematic representation of the initial infection (red thunderbolt) and its progression (red lines and arrows) within a source leaf of an infected plant. Infectious material passes through plasmodesmata (Pd, double green ovals) from mesophyll (ME), bundle sheath (BS), vascular parenchyma (VP) to companion cells (CC) to access sieve elements (SE) and reach the distant tissues. A reverse route occurs in the upper leaves (sink leaf) or roots (not represented). Right panels show leaf venation and illustrate viral phloem loading and unloading through minor and major veins of source and sink leaves. X, Xyleme; I−V: vein classes.

The question about the requirement for the long distance movement of the ‘virus’ of either virion (packaged RNA) or RNP complex is still open and it appears closely linked to the ability of the viral RNAs to be “packaged.” Such infection of vascular tissues is not as uniform as those of mesophyll cells where phytoviruses usually begin their journey by moving cell to cell (for review see [6]). Several boundaries constituted by the bundle sheath (BS) of V or IV class veins (for vein classification, refer to [7]) followed by the vascular parenchyma (VP) have to be passed by the viral material in order to reach companion cells (CCs) and follow the photoassimilates flow of the sieve elements (SEs). To finish the journey, the reverse route passing through class III veins to access CC and then mesophyll cells has to be pursued to start moving cell-to-cell in the distant tissues (Figure 1). 

Whatever the structural shape adopted for plant multipartite RNA viruses, their genomic segments are separated by distinct capsids. This situation raises concerns about the preservation of the viral genome integrity that requires all RNA segments to move from one cell to a neighbor or distant cell in the plant (Figure 1). This ensures the setup of a novel infection site where all viral genes need to be expressed. Here, up to three-decade old as well as new literature describing packaging, cell-to-cell and vascular movement of plant icosahedral and helical viruses has been reviewed to bring into the light our conceptual view of the nature of the systemic moving of viral material in the infected plant. Without addressing details about the RNA expression and replication mechanisms, we propose to distinguish between virions acting as storage of infectious material, ready for transmission, and moving material constituted of RNP complexes containing all RNA segments of the multipartite genome. These RNP complexes involving RNA-RNA interactions act as drivers for the “in planta” viral cycle and appear as the adequate entities for genome preservation, particularly in the long-distance journey of the virus. While this concept appears “insignificant” for monopartite RNA viruses, multipartite viruses need to preserve the expression of their entire genome in the targeted cells. After the description of chosen monopartite phytoviruses, we review and discuss the situation of multipartite RNA viruses and focus on their need to preserve the expression of their entire genome in the targeted cells. A model for benyviruses has been illustrated to present our hypotheses.

## 2. Lessons from Turnip Crinkle Virus, Satellite Tobacco Mosaic Virus, Groundnut Rosette Virus and Tobacco Mosaic Virus: Monopartite RNA Genomes

For some viral species, icosahedral shells are able to assemble in virus-like particles where the genome, usually dsDNA, is incorporated using energy-dependent nanomachines, extensively studied for bacterioviruses T4, P22 or Ø29. The latter uses a non-coding RNA hexamer for DNA entry. Recent insights about the genomic encapsidation of the positive-strand RNA genome of bacterioviruses MS2 revealed the importance of coat protein (CP) dimer interactions, with an estimated number of 60 hairpin RNA structures distributed around the RNA genome (packaging signals), leading to induced-fit RNA-viral protein interactions rather than electrostatic interactions. In this sense, genomic MS2 RNA constitutes a scaffold for assembly initiated or terminated by the assembly protein, following a Hamiltonian path [8,9,10].

### 2.1. Positive-Strand RNA Packaging into Icosahedral Units

#### 2.1.1. Autonomous Turnip Crinkle Virus

Turnip crinkle virus (TCV) from the *Tombusviridae* family is structured by an association of 180 copies of a 38 kDa CP. Its crystallographic structure resembles the tomato bushy stunt virus. TCV viral particles can be dissociated at elevated pH where RNA is attached to six CP subunits that permit reassembly in vitro [11]. Mapping protein-RNA interactions revealed the interaction between CP and two RNA domains. A 15 base-pair (bp) long hairpin structure has been identified within the replicase gene and a 28 nucleotide (nt) bulged hairpin loop is present within a 186 nt essential element within the CP coding sequence. Viral encapsidation was also shown to be dependent on the length of the viral RNA [12], suggesting the requirement of a separation between 5′ (replicase gene) and 3′ (CP gene) CP-interacting sequences, suggesting a bipartite packaging signal for this monopartite RNA viral genome. TCV CP is dispensable for the viral cell-to-cell movement but essential for the systemic spread [13] as this protein acts as a suppressor of RNA silencing [14,15].

#### 2.1.2. Helper Virus Replication-Dependent Satellite Tobacco Mosaic Virus

Satellite tobacco mosaic virus (STMV) is an icosahedral *T* = 1 virus with a small genomic RNA. Atomic structure revealed 30 stem-loop RNA structures, each associated with a two-fold symmetry axis [16]. Interestingly, the RNA secondary structure of STMV analyzed in solution [17] does not correspond to the structure predicted in the *T* = 1 capsid. This discrepancy indicates a selection of the RNA genome into an icosahedral structure following a Hamiltonian pathway as described for MS2 bacteriophage [10]. The existence of multiple RNA folding structures of the genome could account for distinct functions tightly regulating viral protein expression and regulated by viral proteins. This satellite virus requires a helper virus (HV) for its replication. Interestingly, HV replicating STMV belong to helical viruses such as tobacco mosaic virus (TMV) [18]. A systemic movement of both the satellite virus spherical particle and HV helical entities would require a mechanism able to recognize both structural entities. However, drawing hypotheses about possible interactions between satellite RNA and HV RNA could explain a satellite RNA transport by an HV RNP moving complex.

#### 2.1.3. Umbraviruses Replicate Autonomously but Are Packaged in *Trans*

Groundnut rosette virus (GRV) belongs to the *Umbravirus* genus and does not form conventional virions. Indeed, this species does not encode coat proteins. GRV possesses a monopartite genome encoding proteins necessary for its autonomous replication and movement within the plant. Its genome encapsidation occurs during mixed infection with an HV that belongs to the *Luteoviridae* family, specifically transmitted by aphids and only in the presence of a satellite RNA required for the selective encapsidation of GRV [19]. In a host plant, GRV is able to replicate on its own and infect the entire host while the helper luteovirus remains restricted to the phloem. GRV movement is provided by the expression of the open reading frame (ORF) 3 product and recruits fibrillarin to ensure the formation of an RNP complex essential for its systemic spread [20]. This example illustrates the systemic movement of a viral entity without viral shell formation. 

### 2.2. Positive-Strand RNA Packaging into Helical Units: Lessons from Tobacco Mosaic Virus

The reconstruction of TMV from purified CP and viral RNA [21] set up the fundamental basis for understanding rod-shaped and flexuous helicoidal viruses that has been recently reviewed elsewhere [5]. Upon the recognition of an RNA signature on the viral genome, CP nucleates and cooperatively recruits further copies of structural proteins to cover the entire genome with a first coverage of the 5′ domain followed by the protection of the 3′ terminus. Conversely, uncoating of TMV is thought to occur in the cell by a partial disassembly of the CP subunits at the 5′ termini of viral RNA (Ω region) leading to ribosomal scanning and uncoating while the first ORFs are translated. The encoded proteins produce the replicase complex that recognizes the 3′ partially uncoated domain and starts the synthesis of the viral complementary strand and simultaneously removes remaining CP-associated subunits. This ensures a proper recognition of internal promoters required for the subgenomic RNA synthesis and left-ended terminal promoter required for positive-strand synthesis. While subgenomic RNAs (sgRNAs) are produced, expression of the movement protein (MP), required for the efficient transport of viral material from the infected cell to neighboring cells, and CP expression finalize the progression of the viral cycle. TMV CP assembles spontaneously without any cellular factors. TMV genome recognition and encapsidation require both the formation of a 34 to 38 CP subunit disk (namely protohelix or two-ring disk) and the presence of an origin of assembly (OA) on the viral RNA. This leads to the rapid encapsidation (~6 min) of the ~6.4 kb RNA with 2130 copies of the CP subunits. TMV-OA is constituted by three stem loops, with stem loop 1 playing an essential function for the initiation of nucleation, together with a repetition of G residues every three nucleotides within an unpaired region [22]. Importantly, this property led to the engineering of a viral Lego^®^ for nanotechnologies. The combination of genetically modified CP subunits, sometimes together with wild-type CP (both expressed in *Escherichia coli*) with RNAs containing TMV-OA, led to the construction of nanostructures displaying new chemical reactivity, owing to their usage as nanomaterials [23]. 

Reverse genetics using a full-length infectious clone of TMV demonstrated that the CP is not required for cell-to-cell movement but is needed for the efficient systemic infection of hosts such as *Nicotiana benthamiana (N. benthamiana)* [24]. Hence, TMV particles would thereby constitute stable mechanically transmissible entities between sessile hosts whereas RNP complexes containing the CP could represent the moving material within the host. This is illustrated by the description of TMV RNPs isolated from infected *N. tabacum* cv. Samsun plants [25]. Such RNPs differ from virions by their higher buoyant density and contain TMV genomic RNA, polypeptides of 17.5 kDa (TMV CP), 31 kDa (possibly TMV MP), 37 kDa and 39 kDa. Purified RNAs from these RNPs retained the ability to be encapsidated in vitro [25].

The progression of TMV infection within mesophyll cells is well characterized and occurs thanks to the transport of uncoated viral RNAs and MP RNP complex. However, little is known about the nature of the infectious material that permits viral vascular movement. Disruption of the OA by point mutations that preserve functional CP abolishes TMV systemic movement, suggesting that virions represent the long-distance entities [26]. Nevertheless, experimental results outlined below suggest that the hypothesis of TMV virion as the moving material may have to be reconsidered. 

As stated above, in the absence of CP synthesis, TMV is able to move cell-to-cell to access VP but fails to enter CC, suggesting a requirement of the CP to cross the VP/CC boundary [27]. The TMV CP seems to play a central role in the passage of the infectious material through vascular tissues. However, the formation of viral particles does not appear to be a prerequisite because several mutants (insertion or deletion) that affect the RNA binding region of the CP are still able to move long distance, albeit with sometimes delayed kinetics [28]. The crossing of each specific boundary between different cell types seems to require different strategies that could involve dedicated viral factors. 

Masked strain M-TMV shows a delayed systemic spread as compared to the virulent strain U1-TMV. However, comparable replication levels and cell-to-cell movement efficiency in mesophyll cells is described for the inoculated leaves [29]. The delayed systemic movement behavior is attributed to an eight-amino acid mutation in the 126 kDa protein, formerly acting as an RNA silencing suppressor, and hence in the 183 kDa replication protein. Immunohistochemical analysis of class V veins of infected leaves revealed a deficient viral accumulation in the VP and, consequently CC cells, for viruses carrying the p126 mutation [30]. The different behavior of the two strains is maintained in class III veins and mesophyll tissues of non-inoculated leaves even if the number of infected CCs is comparable, suggesting that wild-type p126 is required for BS/VP and CC/VP boundary crossings [30]. It is not known if p126 acts alone or as part of a vRNP complex [30]; however, viral replication complexes constituted of p126, MP and viral RNA are reported to move between cells [31].

Although RNA stabilization by encapsidation may be a prerequisite for TMV to enter VP and CCs, it is not necessary in the SE where no RNase activity was detected in exudates [32,33,34]. Furthermore, the absence of translation could reinforce the hypothesis of a viral RNP complex as the form of transport, because its correct conformation should not be disrupted by the scanning activity of ribosomes. Replacement of the TMV CP by GRV Orf3 leads to systemic movement of the recombinant viral RNA without the formation of virions [35]. The Orf3 product interacts with TMV RNA to form helical-structured filamentous RNP complexes lacking the uniform rod-shaped viral particles [36].

Other indirect experiments permit speculation about the possibility of MP to be included as a constituent of the viral entity that moves long distance. In tobacco plants with a reduced synthesis of pectin methylesterase in vascular tissue, a cell-wall enzyme which interacts with MP [37], TMV is able to load in the SE but cannot be unloaded in non-inoculated leaves [38]. Beyond the CP, the MP is required for TMV cell-to-cell and long-distance movement that could involve two independent mechanisms. Gain and loss-of-function experiments support such a hypothesis. TMV does not systemically infect vanilla orchid. The related *Tobamovirus* odontoglossum ringspot virus protein (ORSV) is able to infect such a host but is unable to move long distance in tobacco [39]. The replacement of TMV MP by ORSV MP permits chimeric TMV to systemically infect vanilla orchid and restrains the infection to local lesions in tobacco plants [39]. The host-range determinant of ORSV resides in the eleven C-terminal amino acids of the MP. The deletion of this domain restores the ability of the chimera to provoke a systemic infection on tobacco and prevents the spread in orchids [39]. 

## 3. Multipartite RNA Genomes

### 3.1. Positive-Strand RNA Packaging into Icosahedral Units

#### 3.1.1. Dianthovirus: Bipartite Genome

Red clover necrotic mosaic virus (RCNMV) belongs to the *Dianthovirus* genus and *Tombusviridae* family. This icosahedral virus possesses a bipartite positive-sense single-stranded RNA genome. RNA1 codes for the proteins involved in replication and also for CP subunits through a sgRNA [40,41,42,43,44]. RNA2 is monocistronic and codes for the MP, a multifunctional protein implicated in suppression of RNA silencing, in cell-to-cell and long-distance movement of the virus [45,46,47]. Although RNA1 replicates alone, it is not able to produce the sgRNA or CP subunits in the absence of RNA2 [48,49,50,51]. A kissing interaction has been found between RNA2 and RNA1. This 8-base pairing occurs between the trans-activating TA element, present in the RNA2 MP ORF and the TA-binding site (TABS) present in sgRNA promoter on the RNA1. The heterodimerization is essential for the production of sgRNA [51], whose production is regulated by RNA2. This interaction is also required for the RNA1-RNA2 packaging [48], because no OA is present on RNA1. Besides these biological roles, the kissing interaction within the OA allowed the engineering of virus-like particles able to efficiently encapsidate nanoparticles or Quantum Dots [52]. Therefore, the particularity of RCNMV is, on the one hand, the encapsidation of the RNA heterodimer in the same particle and, on the other hand, the packaging of multiple copies of RNA2 that could fit in tailored viral shells [53]. 

While the CP is not required for cell-to-cell movement, it is considered necessary for the systemic spread of RCNMV [54]. However, some experiments performed in *N. benthamiana* revealed that RNA1 and RNA2 could move long distance in the absence of the CP and thus without the formation of virions [42]. Although this property appears to depend on the host plant and temperature, it does illustrate again that systemic movement as an RNP complex is possible. Whatever the precise mechanism of its systemic movement, this bipartite viral genome provides direct evidence for the regulation of the viral cycle via the RNA-RNA interaction of distinct viral RNA species.

#### 3.1.2. Brome Mosaic Virus: Tripartite Genome

All three components of the tripartite genome of brome mosaic virus (BMV) (~8.2 kbp) are essential for viral replication and for systemic infection of plants. The RNAs are encapsidated separately in 28 nm particles with *T* = 3 quasi-icosahedral symmetry [55]. RNA1 and 2 are packaged separately, whereas RNA3 is co-packaged with sgRNA (namely RNA4) into a third particle [4]. A ~200 nt conserved 3′ tRNA-like structure (TLS) with determinants for tyrosylation is present on all genomic RNAs and acts as a nucleating element (NE) of CP subunits. This NE is required for the packaging of viral RNA3 and RNA4 [56] but not for RNA1 and 2 packaging [57]. Conserved 3′ untranslated regions (UTRs) appeared to contain additional elements promoting encapsidation and distribution of the RNAs in three indistinguishable particles. The RNA3 encapsidation is dependent on a 187 nt long packaging element (PE) that resides within the MP coding sequence [58]. Besides, RNA3 and 4 packaging was found to be dependent on the replication and on a possible interaction between RNA3 PE and RNA4 NE domains [59]. In wheat germ extracts, pre-swollen particles render viral RNAs available for direct protein synthesis [60], suggesting a co-translational disassembly that has been observed for other spherical viruses such as alfalfa mosaic virus, cowpea chlorotic mottle virus and southern bean mosaic virus [61]. During the course of the viral replication cycle, viral RNAs and proteins allow new viral particles to accumulate. This morphogenesis takes place around replication factories: the co-localization of BMV replicase, CP and viral RNAs could thus control packaging specificity [62,63]. Viral cell-to-cell movement is dependent on the expression of the 3a movement protein, whereas CP is dispensable in some natural or MP-mutated BMV isolates [64,65]. 

Packaging was thought to play an important role in long-distance movement of the virus in the plant. However, using *Agrobacterium tumefaciens*-mediated transient expression of BMV genomic RNAs in leaves of *N. benthamiana*, Gopinath and Kao demonstrated that viral RNAs can also move long distance independently, without the presence of the viral CP [66], probably forming RNP complexes with cellular factors. When produced alone, BMV RNA3 is able to move long distance without the assistance of any viral proteins, while RNA1 and RNA2 both need the MP for systemic movement [66]. This allowed the reconstruction of the entire functional genome in distant tissues of the plant, a requisite for the successful infection of the host. Taken together, these data suggest that the preservation of genome integrity during long-distance movement appears to be mediated by means of a complex in which BMV RNA3 together with MP could play a fundamental role. This led to the hypothesis that viral RNAs interact, in the same cell or in the same environment such as vascular tissues, during the transit of RNA/RNP species. Another aspect of a systemic movement mechanism can be drawn as a result of this experiment. One could argue that in plant, interconnected cells could exchange viral proteins involved in the amplification or in the movement of the viral RNAs that could reach distant cells. In this situation, the proteins or the RNAs will then act in *trans*. To access distant cells, *trans* acting proteins or viral messenger RNAs will be needed in huge amount. Taking these requirements into consideration, only segmented DNA viruses—such as geminiviruses or nanoviruses (circular ssDNA genome) that express mRNA thanks to their stabilized replicative dsDNA segments—can fulfill such a constitutive expression. These viral DNAs ensure a continuous expression of proteins in different tissues, and viral products could move from one cell to another as proteins, mRNAs or both. In the case of BMV described above, the use of agroinfection allowed the continuous production of infectious viral RNAs from cDNAs. This situation recapitulates what could happen for segmented DNA phytoviruses described above.

Recent advances using RNA sequencing (RNAseq) analyses demonstrated the role of TLS structures in the systemic transport of mRNAs containing such structural motifs [67]. The presence of such TLS on BMV RNAs (and TMV as well) may explain why these RNAs could move on their own. Some multipartite RNA viruses such as the benyviruses described below do not possess 3′ TLS and thus will require the presence of a dedicated highly structured motif within each segment to fulfill the movement of each genomic RNA. However, one should take into account that RNA amplification of multipartite RNA viruses depends at least on the expression of the replication machinery and a viral suppressor of RNA silencing. Segregated segments will not be autonomous for amplification and their expression will be transient and not sufficient to promote the production of viral protein or to allow a stabilization of yet non-replicated RNA segments. Therefore, these RNAs will face the RNA decay or could induce cell death if they are expressed. Furthermore, if we assume the movement of viral proteins between communicating cells, this will require the presence of a sequence signal for each protein species for their intercellular transport. A study quantifying the degree of symplastic continuity between plant cells revealed that mobility of non-targeted proteins is strongly dependent on their molecular weight, on the developmental stages and the growth conditions [68]. Thus, if these hypotheses could not be completely excluded, they are difficult to conceive for plant multipartite RNA viruses, particularly for those possessing a helical shape.

### 3.2. Positive-Strand RNA Packaging into Helical Particle Units

If TLS motifs and/or signal peptides could drive the systemic movement of RNP complexes, the hypothesis of an RNP involving RNA-RNA network nucleating genomic segments and driven by few viral proteins appears easier to draw. To highlight our thoughts and findings, we present our views applied to the benyvirus biology and then extend our approach to pomoviruses.

#### 3.2.1. Benyviruses: Up to Five Genomic RNAs 

The *Benyviridae* family is composed of the *Benyvirus* genus of four viral species, namely, beet necrotic yellow vein virus (BNYVV), beet soil-borne mosaic virus (BSBMV), burdock mottle virus and rice stripe necrosis virus, the latter two species having a bipartite genome [69]. Benyviruses are rod-shaped virions of variable length with a constant diameter ranging from 18 to 20 nm. The particle length depends on the genomic RNA individually packaged. Among this family, BNYVV is the most studied species together with BSBMV. These two viruses share a similar genome organization, segment number, host range and transmission vector. Serologically distant, one species is able to amplify and package genomic RNA segments of the other in laboratory conditions [69]. This indicates the presence of conserved promoter structural elements at the extremities of the genomic RNAs, the ability of subgenomic promoter recognition by the replicase, as well as a possibility of RNA-RNA interactions allowing RNP complexes´ formation and function.

**Figure 2:**
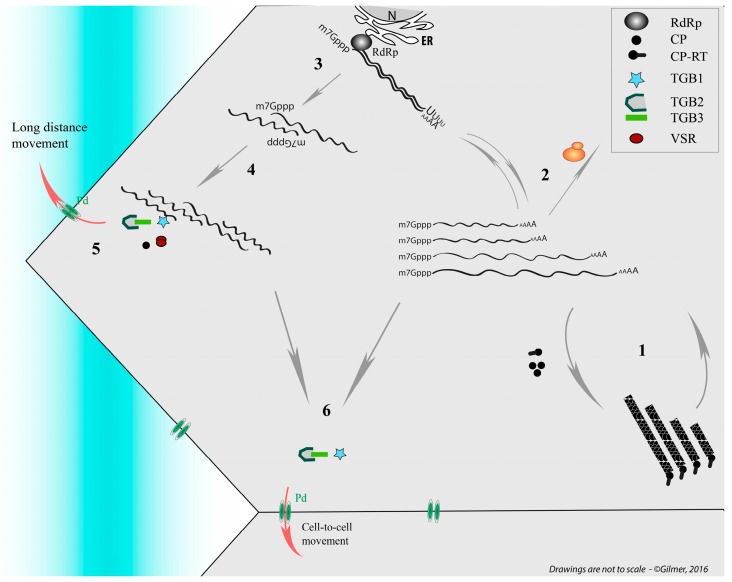
Simplified representation of replication cycle and movement of Beet necrotic yellow vein virus (BNYVV) used as model. The four genomic RNAs of the segmented helical virus (1) are expressed and amplified (2). On this scheme the RNA-RNA interaction network between genomic segments (3 and 4), stabilized by viral proteins, allows the preservation of genome integrity during long-distance movement (5) in the sieve elements (blue shaded). In particular the presence of the coat protein (CP) and the viral suppressors of RNA silencing (VSR) is necessary for their systemic movement. Vice versa the genomic RNAs can move cell to cell without the need of the CP expression but require triple gene block (TGB) movement proteins (6). ER: endoplasmic reticulum; CP-RT: coat protein-readthrough; N: Nucleus; RdRp: RNA-dependent RNA polymerase.

The OA for viral particle morphogenesis has only been studied on the RNA3 and RNA4 species. Preliminary experiments indicate the presence of *cis* acting sequences located in the first 300 and the last 70 nt of the RNA3 species [70]. Since the non coding RNA 3 (former sgRNA3) sequence coterminous with RNA3 is not encapsidated, the OA sequence was presumed to reside within the 5′ UTR of RNA3 [70]. Using a collection of deletion mutants, an encapsidation signal was identified between nt 181–207 [71,72]. The absence of sequence conservation between this RNA OA and other viral RNAs suggests that this domain could be involved in a particular and transient folding required for the packaging. Moreover, nothing is yet known about the possible existence of a bipartite motif that could involve the 3′ conserved UTRs since the RNA3-derived replicon vector (Rep3, constituted by the 5′ and 3′ UTRs of RNA3) is encapsidated [70,72]. The recognition of the viral OA by structural proteins differs from the one described for tobamoviruses (e.g., TMV). To ensure a correct encapsidation process, the minor structural protein needs to be expressed by a read-through bypass of the coat protein stop codon [73]. This 75 kDa coat protein-readthrough (CP-RT) protein is recovered from purified viral particles and has been visualized by transmission electron microscopy (TEM) after immunolabelling [74]. Mutants expressing the CP alone or missing the N-terminal part of the RT domain are deficient in packaging [75,76]. These data suggest a recognition of the viral OAs by the CP-RT domain followed by a cooperative recruitment of the CP subunit to ensure proper packaging [76]. If it exists, the specific subcellular localization of virion assembly is unknown. However, on the one hand, the encapsidation could be coupled to the replication process and thus be associated with the endoplasmic reticulum (ER) vicinity as the replication seems to occur on the ER (Figure 2, step 2) [77]. On the other hand, this packaging could also be transiently associated to mitochondria as virions surrounding the organelle structures have been observed [78]. Further work conducted on the CP-RT protein allowed the identification of a mitochondrial targeting sequence and a transmembrane domain on the minor structural protein [79].

In local host species such as *Chenopodium quinoa*, BNYVV produces local lesions within which each RNA species accumulates and encapsidates individually (Figure 2, step 1) [80]. Local lesion formation requires at least the presence of RNA1 and RNA2 to fulfill the housekeeping functions of replication, cell-to-cell movement and viral suppression of RNA silencing. The coat protein is dispensable for local infection [81].

In systemic hosts such as *Beta* species or *N. benthamiana*, local infection starts as in *Chenopodium quinoa* and then reaches the vasculature of the plant leading to the possible dissemination of the viral material in the entire plant as described in Figure 1. The differential accumulation of virion species in primary infected cells [80] argues against a dissemination of viral particles. On the one hand, taking into account the existence of an unidentified receptor for the virus, there is likelihood to saturate this entry gate with particles containing small genomic RNA species unable to replicate on their own. On the other hand, in a model lacking a receptor for the viral entry, even if still non-estimated, the probability for each segment to enter the same distant cell appears low. Similarly, non-orchestrated dissemination of a viral genomic segment to randomly distant cells will end up with the same conclusion: a low probability to reconstruct the entire viral genome. Our interrogations about the nature of moving material started with the following observations on BNYVV. All viral particles are structurally identical and only differ by their size, and all four (sometimes five) viral genomic RNAs are required to fulfill the entire cycle on the *Beta* species hosts [82,83,84]. However, RNA1 and RNA2 are sufficient for *N. benthamiana* systemic infection thanks to a replication machinery provided, as stated before, by RNA1 and to structural proteins together with the triple gene block (TGB) movement proteins (TGB1, -2 and -3) and post-transcriptional gene silencing suppressor (viral suppressors of RNA silencing, VSR) provided by RNA2 species [85,86] (Figure 2). In this host, RNA3 is dispensable, but when present it moves long distance and accentuates symptom severity [87]. Interestingly, in the presence of RNA1 + RNA2, the RNA3-derived viral expression vector Rep3 does not move long distance in *N. benthamiana* host. As Rep3 is encapsidated, we ruled out the thesis of virions as moving material and we formulated the hypothesis about the existence of RNA-RNA interactions allowing RNA segments to interact with one another to ensure a stabilization of an RNP complex for the long-distance movement of the viral genome in the plant. Such an RNP complex contains CP as this structural protein is required for systemic spread [81]. A reinvestigation of the properties of RT mutants´ behavior described previously [76] confirmed our thoughts. Indeed, in this article some BNYVV mutants unable to form virions are still able to infect plants systemically. Thus, the minimal mobile RNP complex requires RNA1 and RNA2-interacting species to ensure the expression of housekeeping genes. Because RNA2 drives the production of subgenomic RNAs collinearly with its 3′ extremity [88], we postulated that the interaction domain consists of a region present on RNA2 but not included in the sgRNAs sequence, lest 3′ RNA2 collinear sequences interfere with genomic RNA interaction. Therefore, we focused our attention on the first 2000 nt and identified a sequence able to interact with RNA1 species using electromobility shift assay performed on in vitro transcripts. A similar approach performed on RNA3 species revealed the existence of more than one domain involved in the interaction with RNA2 (Dall’Ara et al., in preparation). To confirm our approach and share our findings, we transposed our hypothesis onto potato mop top virus, a tripartite helical virus that does not require structural proteins for its long-distance movement. These findings are discussed below in regard to mutants described in the literature. 

#### 3.2.2. Potato Mop Top Virus: Tripartite, but Two Is Enough

Potato mop top virus (PMTV) belongs to the *Pomovirus* genus together with beet soil-borne virus, beet virus Q, and broad bean necrosis virus. PMTV is a helical rod-shaped virus with a diameter of 18–20 nm and variable lengths (~120 to ~290 nm) according to the genomic ssRNA being packaged [89]. The particles possess a fragile and partially uncoiled extremity [90]. The viral genome is segmented on three positive-sense RNAs. RNA1 allows the expression of two proteins involved in viral replication thanks to a read-through mechanism. RNA2 encodes for major (CP) and minor (CP-RT) structural proteins, the later produced by ribosomal read-through of the CP Amber stop codon. RNA3 possesses four different ORFs that encode for the triple gene block movement proteins (TGB1, TGB2 and TGB3) and one cysteine-rich protein of 8 kDa [89]. As described for BNYVV, minor structural protein is detected at one extremity of some particles. CP-RT is involved in the transmission by the *Spongospora subterranean* vector, because spontaneous deletions occur in isolates with disrupted transmission, particularly after serial mechanical inoculation [91,92]. However, this minor structural protein is dispensable for viral morphogenesis because deletions of the RT domain, or mutations that prevent read-through from occurring, accumulate viral particles [93]. The encapsidation of viral genomic RNAs is also dispensable for systemic spread because “no-protein” and “CP-RT only” RNA2 mutants move [93]. This particularity explains the systemic infection of *N. benthamiana* using a combination of transcripts corresponding to RNA1 and RNA3 [94] and thus demonstrates that PMTV long-distance movement occurs as an RNP complex. The RNA-binding domain constituted by the 84 N-terminal residues of the TGB1 movement protein ensures PTMV RNP complex long-distance movement and appears dispensable for cell-to-cell movement [95]. This domain ensures TGB1 nucleolar localization and its association to microtubules that could allow the recruitment of a cellular factor necessary for the vasculature entry of the RNPs [95]. This renders “PMTV∆RNA2” comparable to GRV, which uses ORF3 protein to recruit fibrillarin to form viral RNP complexes able to move long distance [96] (see Section 2.1.3). 

With the reinterpretation of the literature describing the systemic movement of PMTV RNA2 deletion mutants [93], we pointed out an interesting property for the “CP-only” mutant. This viral RNA2 mutant is unable to move long distance in the presence of RNA1 and RNA3. A similar behavior appears for deletion mutants within the C-terminal part of the CP-RT sequence, leading the authors to conclude that a 140 amino acid CP-RT domain is specifically required for the systemic movement of RNA2. However, abolition of CP synthesis in these mutants restored the RNA2 species´ long-distance transport. Consequently, we postulate on the existence of a sequence acting as a “riboswitch” that could be regulated either by a CP interaction or by refolding during ribosomal translation. Such alternative structures could allow or prevent genomic RNA-RNA network interactions, required for long-distance transport or its recruitment as messenger RNA, respectively.

Taking into consideration our preliminary data accumulated on BNYVV, we checked if similar RNA-RNA interactions could exist, using the IntaRNA program (Freiburg RNA Tools) [97,98] and looking for heterologous RNA-RNA interactions between PMTV genomic RNAs (Sw isolate). We took into consideration the sequences that were preserved in natural and artificial RNA2 deletion mutants that were still able to move long distance [93,99]. We found a 20 nt long sequence (5′- ^2760^UAGGGAAGAUGUCAGGUAAG^2779^ -3′), located within the CP-RT C-terminal coding sequence that could possibly interact with a sequence (5′- ^2244^CUUGCCUGACGUCGAUCCUG^2263^ -3′) present in the RNA1 replicase sequence with a 2 nt bulge (underlined). In a model where genome integrity needs to be preserved during the systemic movement by the association of all genomic RNAs in a RNP complex, such 20 nt base pairing, with a predicted variation of the Gibbs free energy of −16.35 Kcal/mol, could participate in an RNA2-RNA1 specific interaction. We did not extend our computational analyses to the RNA3-RNA2 or RNA3-RNA1 interaction domains. However, taking into account the systemic infection mediated by RNA1 and RNA3, such domains should exist [100].

The advantage of a regulatory network involving RNA-RNA interactions eliminates the need for a viral protein dedicated to associate RNA species with a correct stoichiometry. At the beginning of a cell infection, translation and replication events are predominantly monopolizing viral RNAs that switch between unstructured and structured forms. Thereby, RNA-RNA interactions could take place in *trans* and will guarantee the formation of RNP complexes dedicated to the long-distance movement thanks to the movement proteins produced via subgenomic RNAs. Late in the infection, the accumulation of structural protein would negatively regulate the RNA-RNA interaction, leading to the encapsidation of genomic RNA species ready for their acquisition by the vector thanks to the minor structural protein recognition [92]. As for BNYVV, experiments need to be performed to verify the existence of a “riboswitch” in order to validate or discard these hypotheses. 

## 4. Multipartite Positive-Strand RNA Genomes Have to Preserve Their Genome Integrity

As previously mentioned, to maintain their viral genome as a functional unit, segmented positive-strand viruses require the presence of at least one of each viral RNA segment in the target cell. Such conditions require a high multiplicity of infection (MOI), especially for viruses with four or more genomic entities as demonstrated by theoretical studies [101]. Because the infection of different plant cell types and tissues does not occur as a uniform process, a multipartite phytovirus has to pass through constraints and to sustain other biological costs in order to maintain its genome integrity. 

**Figure 3:**
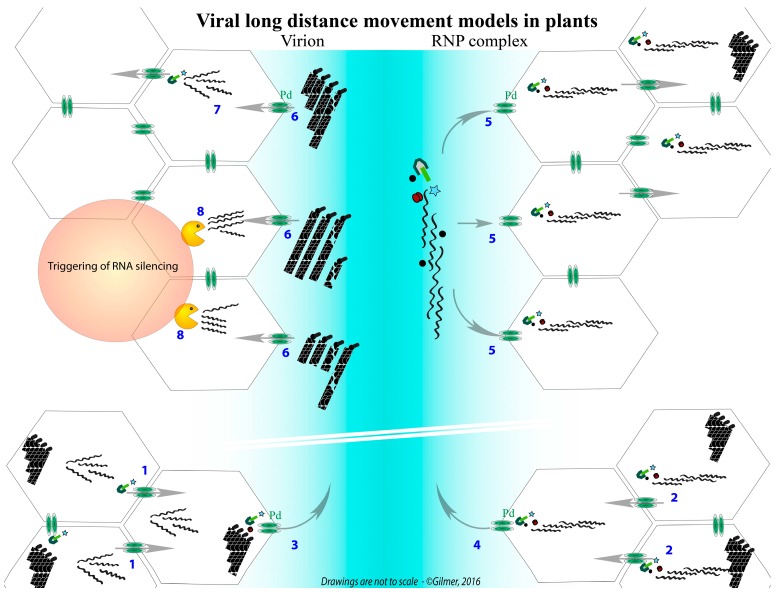
Confrontation of two models describing the long-distance movement of multipartite RNA viruses using the BNYVV as an example. The bottom of the graphic represents the progression of an initial infection of mesophyll cells, wherein the infection progresses thanks to the individual cell-to-cell movement of viral RNAs, driven by TGB movement proteins (1) or as ribonucleoprotein (RNP) complexes involving RNA-RNA network between genomic segments (2). In the sieve elements (blue), the virion (3) or the RNP complexes (4) are transported through plasmodesmata thanks to the viral proteins. RNP complexes, driving the export of the viral genomic RNA network, could provide each distant cell with an entire and fully functional genome (**5**). Virions (or single viral RNAs, not presented) are transported in the sieve elements and are randomly distributed in the distant cells (**6**). Only the combination providing one of each viral particle (or RNA) is able to initiate an infection that preserves the genome integrity (**7**). The particles (or RNA) entering cells without the entire genome are either deficient for replication (not shown) or unable to move from cell to cell and express the VSR, leading to the trigger of RNA silencing (**8**).

Viral cell-to-cell movement ensures the progression of the infection via plasmodesmata, the connection channels between plant cells (Figure 3, steps 1 and 2). In the phloem (a circulation fluid that provides nutrients to all parts of the plant), fluidic mechanic rules dominate. These physical constraints tend to separate small and large viral particles with two tendencies: the first will reduce the velocity of large entities in the fluid, due to increased contacts with the phloem capillary system, whereas small entities will be less affected. Because the phloem is not just a “tube” but consists of vascular elements (sieve elements) connected with companion cells (see Figure 1), viral entities are subject to a “gel filtration” separation. These two effects increase the risk of a loss of one viral component during the journey, compromising the integrity of the viral genome and, therefore, moderate and reduce the speed of systemic movement and infection that should occur in each “visited cell”. 

However, the viral genome must contend with RNA decay mechanisms whereby RNA species are translated or cleaved by the silencing machinery (Figure 3, step 8). Therefore, having its genomic components physically separated would increase the chance of essential viral segments being degraded, thus leading to a defective viral entity. Natural infections show that this is not the case, favoring our idea of a collective transport of genomic RNA species (Figure 3, steps 4 and 5). Hence, RNA-RNA interactions are not the only key elements. Various experiments indicate the involvement of structural protein(s), probably in the stabilization of the interactions, as well as non-structural proteins, such as movement proteins or viral suppressors of RNA silencing that counteract the cellular defenses in the distant cells. 

## 5. Concluding Remarks: Lessons from Superinfection Exclusion Reports

We have reviewed published literature about distinct virus packaging and long-distance movement focusing on some data and arguments in favor of a viral transport as RNP complexes rather than virions. Describing BMV long-distance movement, it appears that the RNA3 species could as well be involved in RNA-RNA interactions with other viral species to nucleate the formation of a RNP complex as postulated for BNYVV and PMTV RNA1-RNA2 species. While one RNA species modulates the recruitment of other species, no information is yet available about the stoichiometry of the genomic components in the moving RNP complex. In order to safeguard genomic integrity, it could be postulated that more than one RNA species should be present to ensure, on the one hand, the expression of essential protein(s) in the distant cell and, on the other hand, the presence of replication-ready genomic components. 

A notable example of preservation of viral genomic integrity is the superinfection exclusion (SIE) that exists among some phytoviruses and prevents some co-infections from occurring. Described for T-even bacteriophages, SIE consists on the inhibition of viral genome entry in the cytoplasm of an infected cell thanks to the expression of viral proteins [102]. In eukaryotes, viral mechanisms are also described for viruses that tend to deplete viral receptors from the infected cell as described for the human immunodeficiency virus (HIV) CD4 receptor internalization and proteolysis thanks to the expression of the Vpu protein [103]. A limited co-infection of distant tissues has been described in plants inoculated with two recombinant monopartite potyvirus variants (Tobacco etch virus) thanks to the use of fluorescent reporter proteins, suggesting as well the establishment of a limited access to the cell, with one variant excluding the other [104]. 

**Figure 4:**
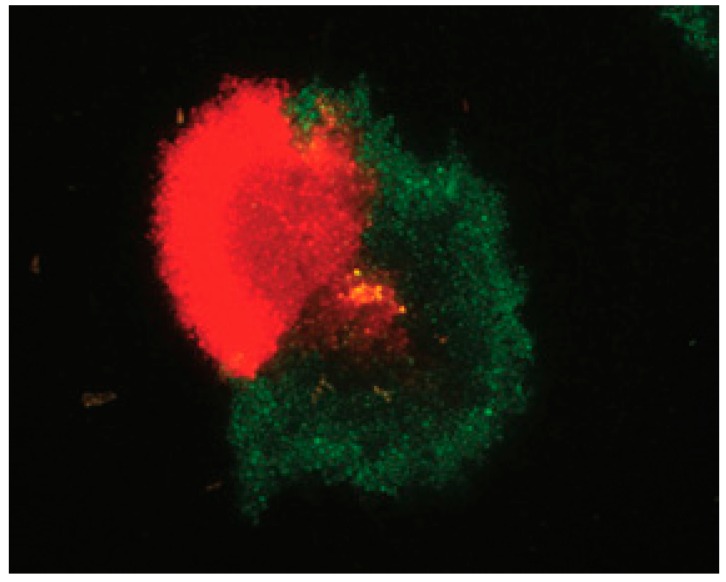
Fluorescence profile of a *C. quinoa* local lesion observed 7 days post infection after rub-inoculation of BNYVV RNA1 and RNA2 supplemented with a mixture of two replicons derived from BSBMV and BNYVV RNA3s, expressing monomeric red fluorescent protein (mRFP) and enhanced green fluorescent protein (EGFP), respectively. This image illustrates the existence of an exclusion mechanism for similar but distinct RNA species during the progression of the infection, with both replicons being able to be encapsidated by BNYVV CP (details are described in Ratti et al., 2009 [104]).

A similar observation was made in sugar beet infected with both BNYVV and BSBMV (two benyviruses). The infection of the plant with one virus limits the possible infection of the same plant with the second virus, if inoculated a few days later [105], suggesting a “SIE-like” mechanism for benyviruses. Using viral reporter RNAs from BSBMV and BNYVV RNA3 replicons, both packaged in *trans* during their amplification by BNYVV RNA1 + RNA2, we previously demonstrated that within local lesions, reporter RNA species exclude one another, leading to sectored lesions as shown in Figure 4 [106]. If such a process occurs on viral particles rather than on viral RNAs, then there will be a high probability for an exclusion of small entities (such as particles containing RNA3 or RNA4) in cells where RNA1 and RNA2 already started to be expressed and replicated. This property bolsters our hypothesis that specific RNA-RNA interactions preserve viral genome integrity during the viral journey within an infected plant. While some plant species susceptible to local infection could resist the systemic spread of these multipartite viruses, it could be assumed that some cellular RNA could compete by interacting with the domains involved in this RNA-RNA network formation, but this is another story…
